# *MicroRNA-200c* Represses IL-6, IL-8, and CCL-5 Expression and Enhances Osteogenic Differentiation

**DOI:** 10.1371/journal.pone.0160915

**Published:** 2016-08-16

**Authors:** Liu Hong, Thad Sharp, Behnoush Khorsand, Carol Fischer, Steven Eliason, Ali Salem, Adil Akkouch, Kim Brogden, Brad A. Amendt

**Affiliations:** 1 Dows Institute for Dental Research, College of Dentistry, the University of Iowa, Iowa City, IA, United States of America; 2 Center for Craniofacial Anomalies Research, Carver College of Medicine, the University of Iowa, Iowa City, IA, United States of America; 3 Department of Anatomy and Cell Biology, Carver College of Medicine, the University of Iowa, Iowa City, IA, United States of America; 4 Department of Pharmaceutical Sciences and Experimental Therapeutics, College of Pharmacy, the University of Iowa, Iowa City, IA, United States of America; Kyoto Daigaku, JAPAN

## Abstract

MicroRNAs (miRs) regulate inflammation and BMP antagonists, thus they have potential uses as therapeutic reagents. However, the molecular function of *miR-200c* in modulating proinflammatory and bone metabolic mediators and osteogenic differentiation is not known. After *miR-200c* was transduced into a human embryonic palatal mesenchyme (HEPM) (a cell line of preosteoblasts), using lentiviral vectors, the resulting *miR-200c* overexpression increased osteogenic differentiation biomarkers, including osteocalcin (OCN) transcripts and calcium content. *miR-200c* expression also down-regulated interleukin (*IL*)*-*6, IL-8, and chemokine (C-C motif) ligand (CCL)-5 under lipopolysaccharide (LPS) stimulation and increased osteoprotegerin (OPG) in these cells. *miR-200c* directly regulates the expression of IL-6, IL-8 and CCL-5 transcripts by binding to their *3’UTRs*. A plasmid-based *miR-200c* inhibitor effectively reduces their binding activities. Additionally, *miR-200c* delivered using polyethylenimine (PEI) nanoparticles effectively inhibits IL-6, IL-8 and CCL-5 in primary human periodontal ligament fibroblasts and increases the biomarkers of osteogenic differentiation in human bone marrow mesenchymal stem cells (MSCs), including calcium content, ALP, and Runx2. These data demonstrate that *miR-200c* represses IL-6, IL-8 and CCL-5 and improves osteogenic differentiation. *miR-200c* may potentially be used as an effective means to prevent periodontitis-associated bone loss by arresting inflammation and osteoclastogenesis and enhancing bone regeneration.

## Introduction

It has been reported that approximately half of American adults aged 30 years and older have periodontitis, and the prevalence of periodontitis further increase in aged populations and in patients with diabetes or who smoke [1, 2]. Approximately 50% of periodontitis patients aged 30 years and older have alveolar bone loss that eventually may lead to tooth loss and osseointegration failure of dental implants, if patients do not receive efficient therapeutics to arrest the progression of this chronic disease [2, 3]. Although anti-resorptive and anabolic agents, including vitamin D, calcium, hormone replacements, and bisphosphonates, are currently used to prevent and treat systemic osteoporosis, their efficacy to arrest periodontal bone loss and improve osseointegration of dental implants has not been confirmed [4–6]. Long-term use of intravenous bisphosphonates has been shown to cause osteonecrosis of the jaw [7].

While bacteria-derived factors initiate periodontitis, there is strong evidence that the majority of periodontitis occurs due to activation of host-derived immune and inflammatory defense mechanisms. Toll-like receptors (TLRs) are the major cell-surface initiators of inflammatory responses to pathogens. TLR-2 and TLR4 play critical roles in recognizing periodontal pathogens and trigger the up-regulation of interleukin (IL)-6, IL-1β, and tumor necrosis factor (TNF)-α in periodontitis [8–10]. TLR-mediated signaling pathways also lead to activation of nuclear factor kappa-light-chain-enhancer of activated B cells (NF-κB), a key proinflammatory transcription factor [11]. These cytokines and transcription factors in turn further amplify the inflammatory response and lead to production of lytic enzymes and stimulate the production of chemokines, including IL-6, IL-8 and CCL-5 [8–10, 12]. Eventually, a cascade of events leads to osteoclastogenesis and subsequent bone resorption via the receptor activator of nuclear factor kappa-B ligand (RANKL)-osteoprotegerin (OPG) axis. Thus, imbalance and dysregulation of proinflammatory molecules and cytokine networks play essential roles in the process of periodontitis and associated bone resorption [8, 9]. Reducing the expression and activation of proinflammatory and bone metabolic mediators that activate osteoclastogenesis and bone resorption may serve as an effective strategy to prevent and arrest the development of periodontal bone loss. Additionally, proinflammatory mediators have been demonstrated to impair bone formation by reducing differentiation of osteoblasts and their progenitor cells [13–18]. Specifically, TNF-α, and IL-1β have been demonstrated to inhibit osteogenic differentiation of bone marrow stem cells. TNF-α also inhibits *Osterix* expression and promotes Runx2 degradation. TNF-α and IL-17 activate IκB kinase (IKK)-NF-κB to reduce osteogenic differentiation of MSCs and impair bone formation by promoting β-catenin degradation. Thus, inhibiting proinflammatory mediators may prevent and restore periodontitis-associated bone loss.

MicroRNAs (*miRs*), non-coding small RNAs, actively participate in inflammation by directly degrading and/or silencing the transcription of targeted genes [19–20]. *miRs* also regulate osteogenic differentiation and bone homeostasis [21]. *miR-200c*, a member of the *miR-200* family, regulates the mesenchymal-to-epithelial transition (MET) [22] and stem cell proliferation and differentiation [23]. *miR-200c* is significantly downregulated in gingival tissues of periodontitis patients [24] and has been demonstrated to participate in signal pathways mediated by multiple proinflammatory factors and repress the expression and activity of NF-kB [24–27]. In addition, *miR-200c* has been found to effectively inhibit Noggin, an antagonist of BMP signals, by directly targeting the *3’UTR* of Noggin [28]. This evidence strongly suggests that *miR-200c* may possess the molecular function to both improve osteogenic differentiation and repress periodontitis-associated proinflammatory cytokines.

In this study, we investigated the molecular effects of overexpressed *miR-200c* using lentiviral vectors on periodontitis-associated proinflammatory factors and the biomarkers of osteogenic differentiation in human embryonic palatal mesenchyme (HEPM) cells, a cell line of preosteoblasts. We found that overexpression of *miR-200c* in the human preosteoblast cell line effectively suppresses multiple proinflammatory mediators, including IL-6, IL-8, and CCL-5, and increases OPG (an osteoclastogenesis inhibitor) and osteocalcin (OCN) and calcium content. Additionally, we used polyethylenimine (PEI), a non-viral nanoparticle delivery system, to successfully deliver plasmid DNA containing *miR-200c* into primary human periodontal ligament fibroblasts and bone marrow MSCs. *miR-200c* delivered using PEI effectively inhibited IL-6, IL-8, and CCL-5 in periodontal ligament fibroblasts and enhanced osteogenic differentiation of human bone marrow MSCs *in vitro*. We reported that *miR-200c* directly targets the *3’UTR* of IL-6, IL-8 and CCL-5. These data indicate the usefulness of *miR-200c* in prevention and restoration for periodontitis-induced bone loss, with the ability to modulate inflammation and bone formation.

## Materials and Methods

### Materials

Plasmids, including psPAX2, pMD2G, and those carrying *miR-200c*, scrambled *miRs*, or the empty vector were purchased from Addgene (Cambridge, MA, USA). HEK 293T and HEPM cells were purchased from ATCC (Manassas, VA, USA). The *miR* inhibitor plasmids were purchased from NaturemiRI (NaturemiRI.com). Primary human bone marrow MSCs and periodontal ligament fibroblasts were purchased from StemCells (Newark, CA, USA) and ScienCell Research Laboratories (Carlsbad, CA, USA), respectively. Taqmen probe and primers for real-time PCR and Sybre Green primers analysis were purchased from Life Technologies and Invitrogen (ThermoFisher Scientific, Waltham, MA, USA). miRNeasy Mini Kits were purchased from QIAGEN (Valencia, CA, USA). Measurements of ALP and calcium were made using kits purchased from AnaSpec (Fremont, CA, USA) and Cayman Chemical (Ann Arbor, MI, USA). All other chemicals and media were purchased from Invitrogen.

### Preparation of lentiviral vectors carrying *miR-200c*

Lentiviral vectors carrying plasmid *miR-200c* or scrambled *miRs* were produced by transfecting *psPAX2*, *pMD2G*, and plasmid carrying *miR-200c* or scrambled *miRs* into HEK 293T cells using a standard CaCl_2_ method. Briefly, 1.8 μg of *psPAX2*, 1.2 μg of *pMD2G*, and 4.2 μg of plasmid *miR-200c* or scrambled *miRs* were mixed with 14 μl of 2M CaCl_2_, and 2 μl of 10mg/ml polybrene in HBS buffer (pH 7.05) to constitute the transfection solution. The transfection solution was then applied to culture plates containing HEK 293T cells at 20–30% confluence, and replaced with fresh medium after 24 hours. Supernatant containing the *miR-200c* lentivirus was then harvested after 72 hours and filtered through a 0.45-μm sterile syringe.

### Transduction of HEPM cells with *miR-200c* using lentivirus

In order to transduce HEPM cells with plasmid *miR-200c* or scrambled *miRs*, the lentiviral vector carrying *miR-200c* (about 10^8^ TU/ml) was added to a suspension of HEPM cells in a 6-well plate and incubated overnight. The medium was replaced each day for 3 days with fresh medium containing the same amount of lentivirus carrying *miR-200c* or scrambled *miRs*. The cells were then collected and sorted by flow cytometry for the presence of green fluorescent protein. The positive cells were analyzed for the proliferation, osteogenic capacity, and proinflammatory mediators.

### Analysis of proliferative and osteogenic capacity of HEPM cells

HEPM cells infected with *miR-200c* or scrambled *miRs* were cultured in Dulbecco's Modified Eagle Medium (DMEM) containing 10% fetal bovine serum (FBS). To determine the proliferation rate, the cells were placed in 6-well plates at 10^4^ cells/per well and collected after 24, 48 and 72 hours. The doubling time of the cells was measured using the equation: Doubling Time = duration * log (2) / log (Final concentration)—log (Initial concentration). To analyze osteogenic capacity, the cells were cultured 6-well plates at 10^5^ cells/per well with osteogenic medium consisting of DMEM supplemented with 1mM β-glycerophosphate and 0.05 mM ascorbic acid-2-phosphate, for up to 2 weeks. The cells transduced with the scrambled *miRs* served as controls. Transcripts of *OCN* were quantified using real-time PCR and calcium content was quantified using Calcium Assay Kits (Cayman, Ann Arbor, MI). Primers and probes were designed using the Primer Express software (Applied Biosystems, Foster City, CA, USA). The primers for OCN were forward: 5”-TAG TGA AGA GAC CCA GGC GC-3”, reverse: 5”-CAC AGT CCG GAT TGA GCT CA-3”, and real-time probes: TGT ATC AAT GGC TGG GAG CCC CAG.

### Construction of *miR* expression and inhibitor plasmids

In order to construct an *miR* expression plasmid, *miR* genes were PCR amplified that include approximately 100bp upstream and 100bp downstream sequence flanking the approximately 80bp stem loop sequence. The PCR product was ligated into pSilencer 4.1 vector (Ambion) digested by BamHI and HindIII. The *miR inhibitor plasmids* was purchased from NaturemiRI (NaturemiRI.com). The construction was established according to our previous studies [29]. Briefly, to construct different designs of *miR inhibitors* for *miR-200c* and *miR-17-18*, we annealed and ligated the *miR-200c* or *miR-17-18* binding sites with a central bulge flanked by different sequences into pLL3.7 vector (Addgene) digested with HpaI and XhoI. To construct the *miR* inhibitor clone vector, we replaced the *miR-200c* binding site with two BsmBI sites in the most effective inhibitor design. AscI and PmeI sites were inserted between ApaI and XbaI sites before the U6 promoter. An SmaI site was inserted before XhoI after the polIII terminator. This vector is termed PMIS-empty vector (EV) for plasmid of *miR* inhibitor. After digestion by BsmBI, pmiRi can be used to clone different *miR* inhibitors into it after annealing and ligation of different miR binding sites with a central bulge.

### Transfection of *miR-200c* using PEI into primary human cells

Plasmid DNA containing *miR-200c* was incorporated into PEI to form nanoplexes at an N/P ratio of 10:1 {ratio of the total number of end amine groups (N) of PEI and the total number of DNA phosphate groups (P)} according to our previous studies [30]. Briefly, the complexes were prepared by adding 50μl PEI solution to 50μl *miR-200c* (10μg) solution and mixed for 30 seconds. The mixture was then incubated at room temperature for 30 minutes to allow complex formation between the positively charged PEI and the negatively charged plasmid DNA. The encapsulation efficiency and plasmid *miR-200c* condensation within the complex were elucidated using spectrophotometry and gel electrophoresis, respectively as in our previous studies [30]. In order to test the transfection efficiency, primary human periodontal ligament fibroblasts and bone marrow MSCs cultured with DMEM medium were seeded in 6-well plates at 10^5^ cells/well. PEI-*miR-200c* nanoplexes at different doses (1, 2, 5, and 10μg/per well) were added into the medium of cultured cells. The medium was exchanged after 4 hours to remove extra nanoplexes and the cells with different treatment were continuously cultured using DMEM medium. After 48 hours the cells with different treatment were harvested using Trizol. Total RNA was collected using miRNeasy Mini Kit and cDNA was prepared using the TaqMan microRNA reverse transcription kit. *miR-200c* transcripts were detected using real-time PCR. The primers and probes of *miR-200c* were purchased from Life Technologies.

### Measurement of proinflammatory mediators

The HEPM cells with *miR-200c* or scrambled *miRs* were placed in DMEM medium at 10^6^ cells/per 25cm^2^ tissue culture flask. For periodontal ligament fibroblasts, the cells were placed in DMEM medium at 10^5^ cells/per well and cultured in 6-well plates and subsequently treated with PEI-*miR-200c* at different concentrations for 4 hours. The cells treated with the same amount of PEI-empty vector severed as controls. The cells were then cultured in DMEM medium for 48 hours. In order to determine the proinflammatory mediators in HEPM cells and periodontal ligament fibroblasts, the cells were cultured using DMEM with or without lipopolysaccharide (LPS) supplement (1μg/ml) (Lonza N-185). To measure the transcript of proinflammatory mediators, the cells with different treatments were collected after 24 hours. Quantitative real-time PCR was used to measure the transcripts of IL-6, IL-8, and CCL-5. The forward and reverse primers for IL-6 were: 5'- CCA TCT TTG GAA GGT TCA GGT TG -3' and 5'- ACT CAC CTC TTC AGA ACG AAT TG -3'. The primers for IL-8 were: 5'- AAC CCT CTG CAC CCA GTT TTC -3' and 5'- ACT GAG GAT TGA GAG TGG AC -3'. The primers for CCL-5: 5'- TGC CCA CAT CAA GGA GTA TTT -3' and 5'- CTT GCT GTC CCT CTC TCT TTG -3'. To measure the proinflammatory mediators in the supernatant of culture medium, a small portion of culture medium (300μl) from different cells with different treatments was collected at different time points up to 32 hours. Chemokine and cytokine concentrations were measured in cell supernatants using Milliplex immunoassays (Millipore, Billerica, MA USA) as previously described [31]. Cell supernatants (25μl) were incubated with anti-human multi-cytokine magnetic beads at 4°C for 18 hours before removing unbound material using a magnetic plate washer (ELx405TS, BioTek, Winooski, VT, USA). Samples were then incubated with anti-human multi-cytokine biotin reporters for *IL-6*, *IL-8*, and *CCL5* for one hour at room temperature, streptavidin-phycoerythrin was added, and plates were incubated for an additional 30 minutes. Samples were washed and suspended in sheath fluid before analysis using a Luminex 100 (Austin, TX, USA). Standard curves for each cytokine were prepared from 3.20 to 10,000 pg/ml and concentrations of chemokines and cytokines in each sample were interpolated from standard curves (xPonent v3.1, Luminex, Austin, TX USA; MILLIPLEX Analyst v5.1, Millipore, Billerica, MA, USA). Additionally, we measured IL-6 and IL-8 of non-treated HEPM cells and compared to the cells with *miR-200c* or scrambled miRs to determine the proinflammatory mediators of HEPM cells varied by *miR* transfection using lentiviral vectors. Non-treated HEPM cells and the cells with scrambled *miRs* or *miR-200c* were treated with DMEM medium supplemented with LPS at 0, 1, 5, and 10 μg/mL. The transcripts of IL-6 and IL-8 in different cells were measured using real-time PCR after 24 hours.

### Analysis of osteogenic differentiation of human bone marrow MSCs

Human bone marrow MSCs were placed in DMEM medium at 10^5^ cells/per well in 6-well plates and subsequently treated with PEI-*miR-200c* at 1μg/per well for 4 hours. The cells were cultured with DMEM medium supplemented with 1mM β-glycerophosphate and 0.05 mM ascorbic acid-2-phosphate, for up to 2 weeks. After the cells were collected and sonicated, the protein level in the lysate was measured using a protein assay kit (Pierce@BCA Protein Assay Kit, Thermo Scientific, Waltham, MA, USA). Biomarkers of osteogenic differentiation, including *ALP* and calcium, were quantified using SensoLyte^®^ pNPP Alkaline Phosphatase ELISA Assay Kit *Colorimetric* (AnaSpec Inc) and Calcium Assay Kit (Cayman Chemical). Transcripts of *ALP* and *Runx2* were quantified using real-time PCR. The forward and reverse primers and real-time probes for *Runx2* were: 5’-CAA CAA GAC CCT GCC CGT-3’, 5’-TCC CAT CTG GTA CCT CTC CG-3’, and 5’-CTT CAA GGT GGT AGC CC-3’. Primers set for *ALP* were 5’-AGC TGA ACA GGA ACA ACG TGA-3’ and 5’-CTT CAT GGT GCC CGT GGT C-3’.

### Luciferase reporter assays

*miR-200c* was cloned into *pSilenser 4*.*1*(Life Technologies). Luciferase reporters were generated by inserting *3’UTR* DNA fragments into *pGL3 CXCR4* vector (Addgene). All the cloned constructs were confirmed by DNA sequencing. All plasmids used for transfection were purified by double-banding in CsCL. Luciferase, Bgal and protein concentration assay were done as previously described [28]. HEPM cells were cultured in DMEM supplemented with 5% FBS, 1% penicillin/streptomycin, and transfected by electroporation. Cells were fed and seeded in 60 mm dishes 24 hrs prior to transient transfection. Cells were resuspended in PBS and mixed with 2.5μg of expression plasmid, 5μg of reporter plasmid and 0.2μg of SV-40 β-galactosidase plasmid. Transfection was performed by electroporation at 380v and 950μF (Gene Pulser XL, Bio-Rad, Hercules, CA, USA), or using the Lipofectamine 200 transfection reagent (Life Technologies). Transfected cells were incubated in 60mm culture dishes, for 24 hrs unless otherwise indicated, and fed with 10% FBS and DMEM. Following lysis, assays for reporter activity (Luciferase assay, Promega, Madison, WI, USA) as well as for protein content (Bradford assay, Bio-Rad) were carried out. β-galactosidase was measured using the Galacto-Light Plus reagents (Tropix Inc., Bedford, MA, USA) as an internal normalizer. For each assay, all luciferase activity was normalized to the mean value of the first experimental group.

### Statistical Analysis

All quantitative data were calculated as means ± standard deviation. The osteogenic differentiation biomarkers and proinflammatory mediators in HEPM cells, human periodontal ligament fibroblasts, and MSCs with overexpressing *miR-200c* were analyzed by one-way ANOVA with Fisher's LSD post hoc test, using commercially available statistics software (SPSS Inc., Chicago, IL), and *p* values fewer than 0.05 were considered significant. Each experiment was performed in triplicate.

## Results

### *miR-200c* expression doesn’t affect cell morphology and proliferation

**Fig 1:**
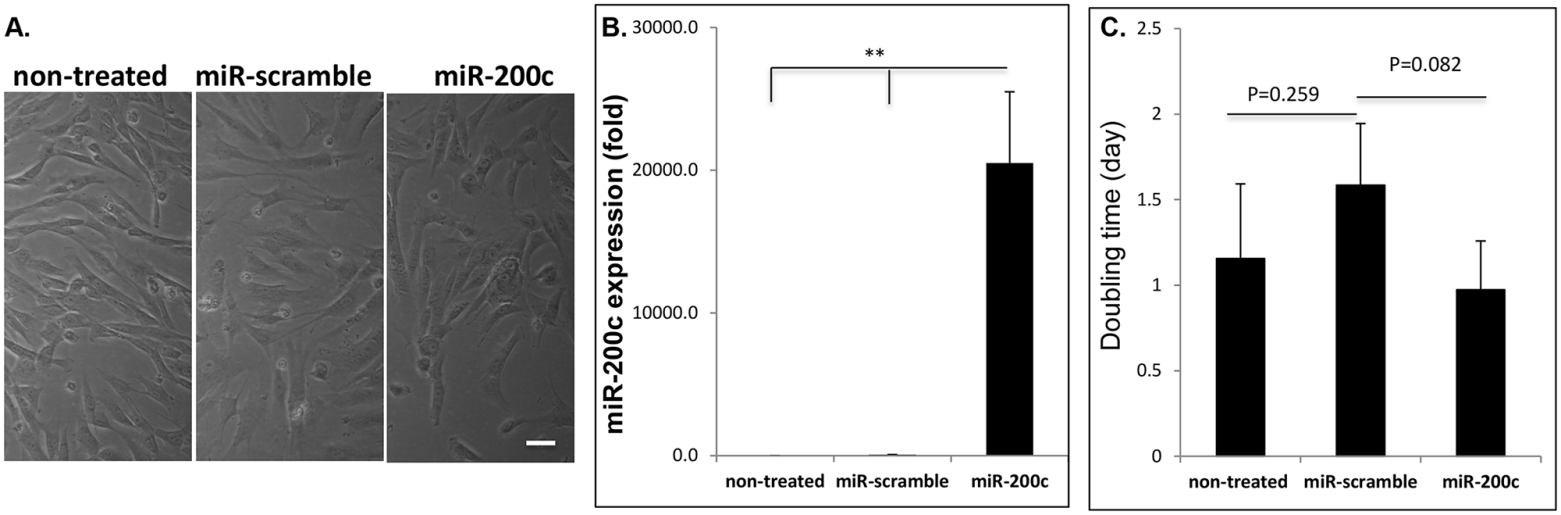
*miR-200c* overexpression in HEPM cells and the effects on their proliferation. **A**: Microphotographs of HEPM cells and the cells with *miR-200c* or scrambled *miRs* under phase-contrast. Bar = 10μm. **B**: Fold change of *miR-200c* expression in non-treated HEPM cells and the cells with *miR-200c* and scrambled *miRs*. **C**: The doubling time of non-treated HEPM cells and the cells in the context of *miR* infection. **: p<0.01.

Lentiviral vectors were used to transfect *miR-200c* into HEPM cells. The HEPM cells overexpressing *miR-200c* maintained a fibroblastic morphology compared to the non-treated cells and the cells with scrambled miRs (Fig 1A). The level of *miR-200c* expression measured using real-time PCR was approximately 2x10^4^-fold higher in HEPM cells infected with *miR-200c* than in non-treated control cells and the cells transfected with scrambled *miRs*, while there was limited *miR-200c* expression in non-treated HEPM cells and the cells with scrambled *miRs* (Fig 1B). The doubling time for the cells with scrambled *miRs* was similar to non-treated HEPM cells, and *miR-200c*-infected HEPM cells did not differ significantly from that in either untreated cells or cells infected with scrambled *miRs* (Fig 1C).

### *miR-200c* modulates proinflammatory mediators and osteogenic differentiation in preosteoblasts

**Fig 2:**
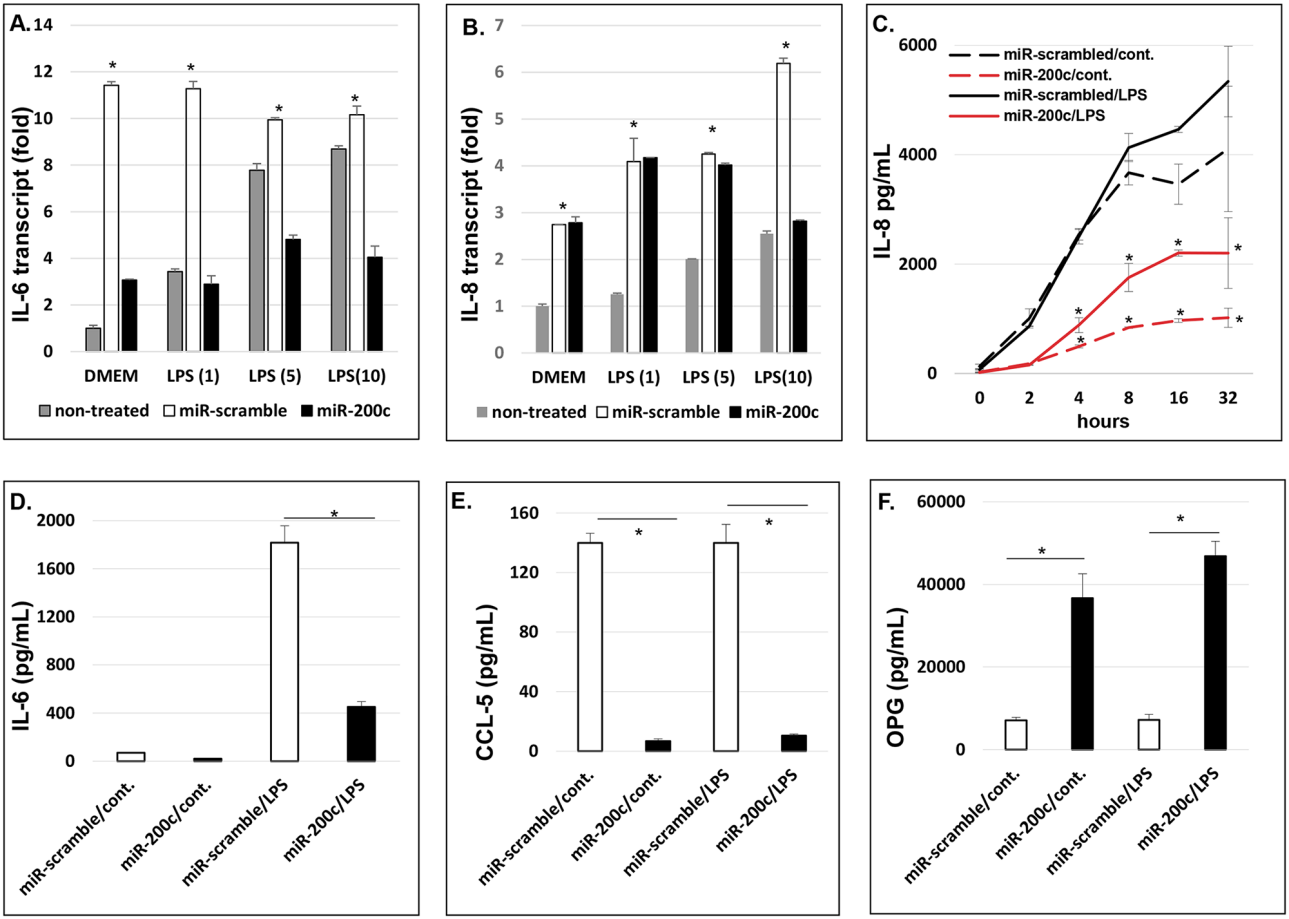
*miR-200c* modulates proinflammatory mediators in human preosteoblasts. **A** and **B:** the transcripts of IL-6 **(A)** and IL-8 (**B**) in non-treated HEPM cells and the cells with *miR-200c* or scrambled *miRs* cultured in DMEM supplemented with LPS at 0, 1, 5 and 10 μg/mL after 24 hours; *:p<0.05 vs non-treated; **C:** the amounts of IL-8 secreted by HEPM cells with *miR-200c* or scrambled *miRs* cultured in DMEM supplemented with or without LPS at different time points; *: p<0.05 vs cells with scrambled miRs; **D** and **E:** the amounts of IL-6 (**D**) and CCL-5 (**E**) secreted by HEPM cells with *miR-200c* or *scrambled miRs* cultured in DMEM supplemented with or without LPS after 24 hrs; **F:** the amounts of OPG secreted by HEPM cells with different *miRs* cultured in DMEM supplemented with or without LPS after 32 hours. *: p<0.05.

**Fig 3:**
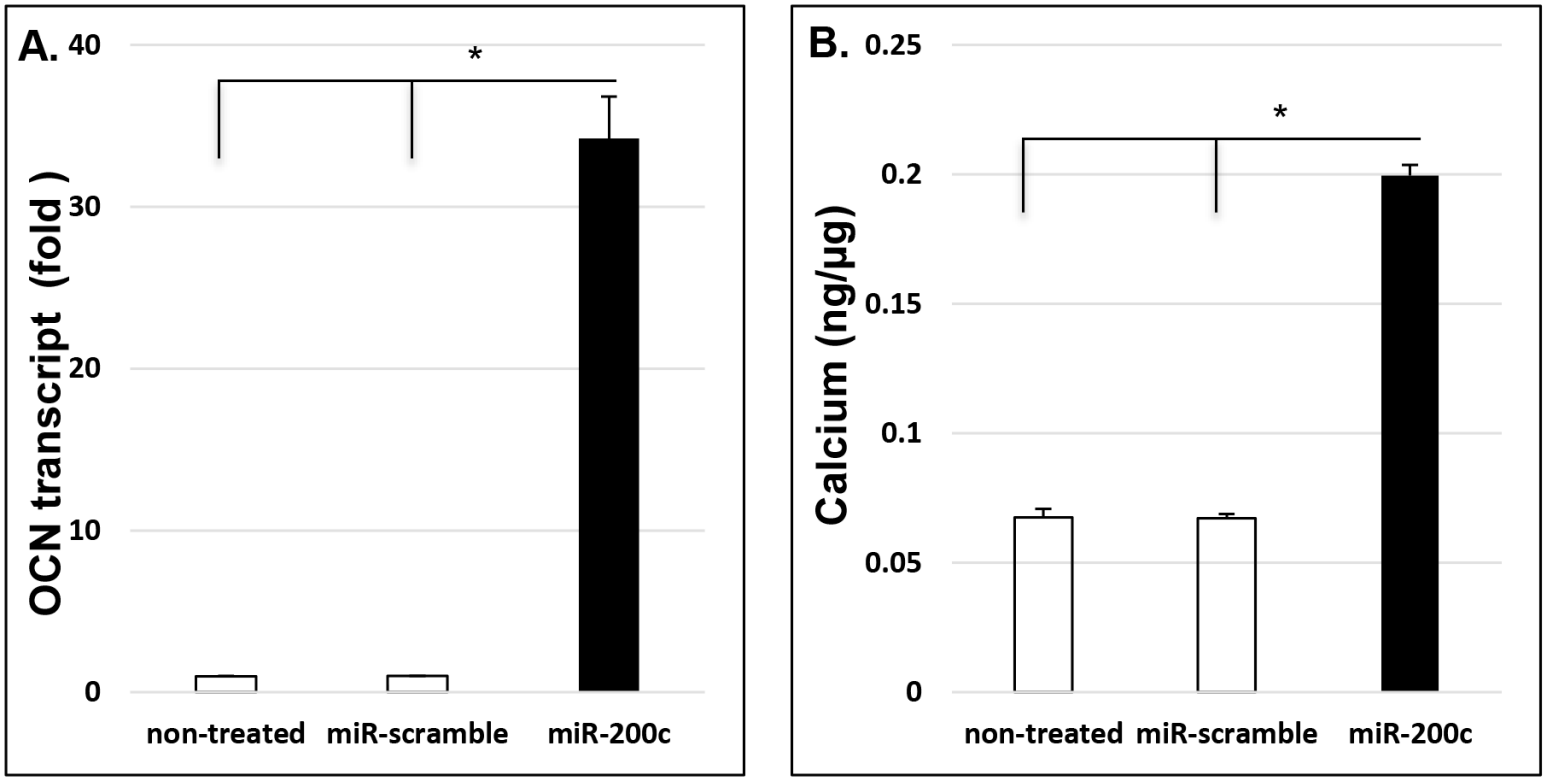
*miR-200c* increases osteogenic biomarkers in human preosteoblasts. **A** and **B**: the amounts of the transcript of OCN (**A**) and calcium content (**B**) in non-treated HEPM cells and the cells with *miR-200c* or scrambled *miRs* cultured in DMEM supplemented *β*-glycerophosphate and ascorbic acid after 1 and 2 weeks, respectively. *: p<0.05.

Fig 2A and 2B summarize the transcripts of IL-6 and IL-8 in non-treated HEPM cells, compared to the cells with scrambled *miRs* or *miR-200c*. IL-6 and IL-8 in the cells with scrambled *miRs* were significantly higher than non-treated HEPM cells after LPS treatment at different concentrations (Fig 2A and 2B). This indicates that transfection of *miRs* using lentiviral vectors may affect the proinflammatory mediators of HEPM cells. Therefore, comparison between HEPM cells transfected with *miR-200c* to the cells with scrambled *miRs* would be more reliable and accurate to determine the function of *miR-200c* overexpression in HEPM cells. We measured the protein concentration of proinflammatory mediators in the supernatant of HEPM cells after HEPM cells transfected with *miR-200c* or scrambled *miRs* were cultured with or without LPS supplement (1μg/ml) up to 32 hours. The amounts of IL-8 in the culture medium of the cells with *miR-200c* overexpression were significantly lower than cells with scrambled *miRs* at each time point (Fig 2C). Cells were cultured with DMEM containing FBS, which contains cytokines and growth factors; however, we measured relative concentrations of IL-8, IL-6 and CCL-5 compared to controls cells also cultured in FBS. LPS supplement increased the amount of IL-8 in HEPM cells with scrambled miRs starting after 4 hours. However, the cells with *miR-200c* produced much less IL-8 than that of controls even after they were exposed to LPS (Fig 2C). Similarly, the amount of IL-6 secreted by the cells with *miR-200c* overexpression in culture medium was lower (3–4 fold) than that of cells with scrambled *miRs* after 24 hours (Fig 2D). With LPS treatment, IL-6 concentrations in the media are significantly increased, the IL-6 concentration of cells with *miR-200c* were significantly lower (2–3 fold) than that of cells with scrambled *miRs* (Fig 2D). In addition, although LPS treatment didn’t effectively increase CCL-5 production in HEPM cells, the cells with overexpression of *miR-200c* produced significantly lower CCL-5 (approximately 20-fold) than the cells with scrambled *miRs* (Fig 2E). The amount of OPG secreted by the cells with *miR-200c* overexpression with or without LPS supplement were higher (6–8 folds) than that of cells with scrambled *miRs* after 32 hours (Fig 2F). After the non-treated HEPM cells and the cells with *miR-200c* or scrambled *miRs* were cultured in DMEM medium supplemented with *β*-glycerophosphate and ascorbic acid, we observed that OCN transcripts measured using real-time PCR in cells with *miR-200c* were significantly higher than the non-treated HEPM cells and the cells with scrambled *miRs* after one week (Fig 3A). The calcium content in *miR-200c* cells was 3 times higher than that of non-treated cells and the cells with scrambled *miRs* after two weeks **(**Fig 3B). There was no difference of OCN and calcium content between non-treated HEPM cells and the cells with scrambled miRs (Fig 3A and 3B).

### PEI nanoparticles deliver *miR-200c* to primary human bone marrow MSCs and periodontal ligament fibroblasts

**Fig 4:**
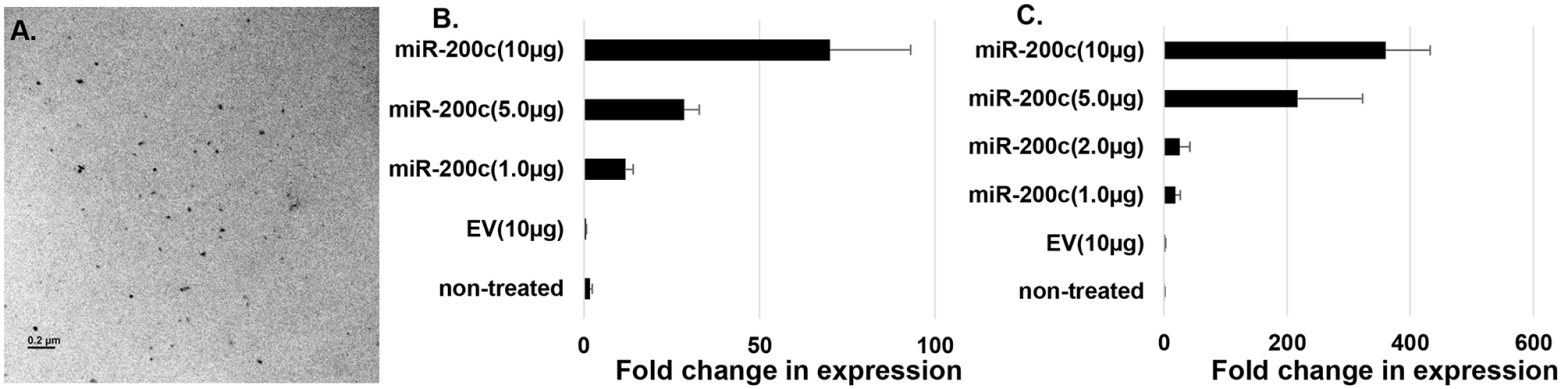
Intracellular delivery of *miR-200c* using PEI nanoparticles to human primary periodontal ligament fibroblasts and bone marrow MSCs. **A**: TEM image of PEI-*miR-200c* nanoplexes. **B** and **C:** Fold change of the transcript of *miR-200c* in non-treated human periodontal ligament fibroblasts (**B**) and bone marrow MSCs (**C**) and the cells transfected with empty vector (EV) (10μg/per well) and *miR-200c* (1, 5, 10μg/per well).

Plasmid *miR-200c* was incorporated into PEI to form nanoplexes at an N/P ratio of 10:1. PEI-*miR-200c* nanoplexes were visualized using transmission electron microscope (TEM) (Fig 4A). PEI-*miR-200c* nanoplexes at 1, 2, 5, and 10 μg/per well were added to the medium of cultured primary human bone marrow MSCs and periodontal ligament fibroblasts in 6-well plates. PEI-empty vector (10 μg/per well) was used as a control. The medium was changed after 4 hours to remove excess nanoplexes and the cells were continuously cultured in DMEM medium. After 2 days *miR-200c* expression was detected using real-time PCR in periodontal ligament fibroblasts (Fig 4B). *miR-200c* dose-dependent expression was also observed in human bone marrow MSCs (Fig 4C). The expressions of *miR-200c* in non-treated periodontal ligament fibroblasts and bone marrow MSCs were similar to that of the cells treated with empty vector.

### *miR-200c* delivered using PEI nanoparticles inhibits IL-6, IL-8, and CCL-5 in periodontal ligament cells

**Fig 5:**
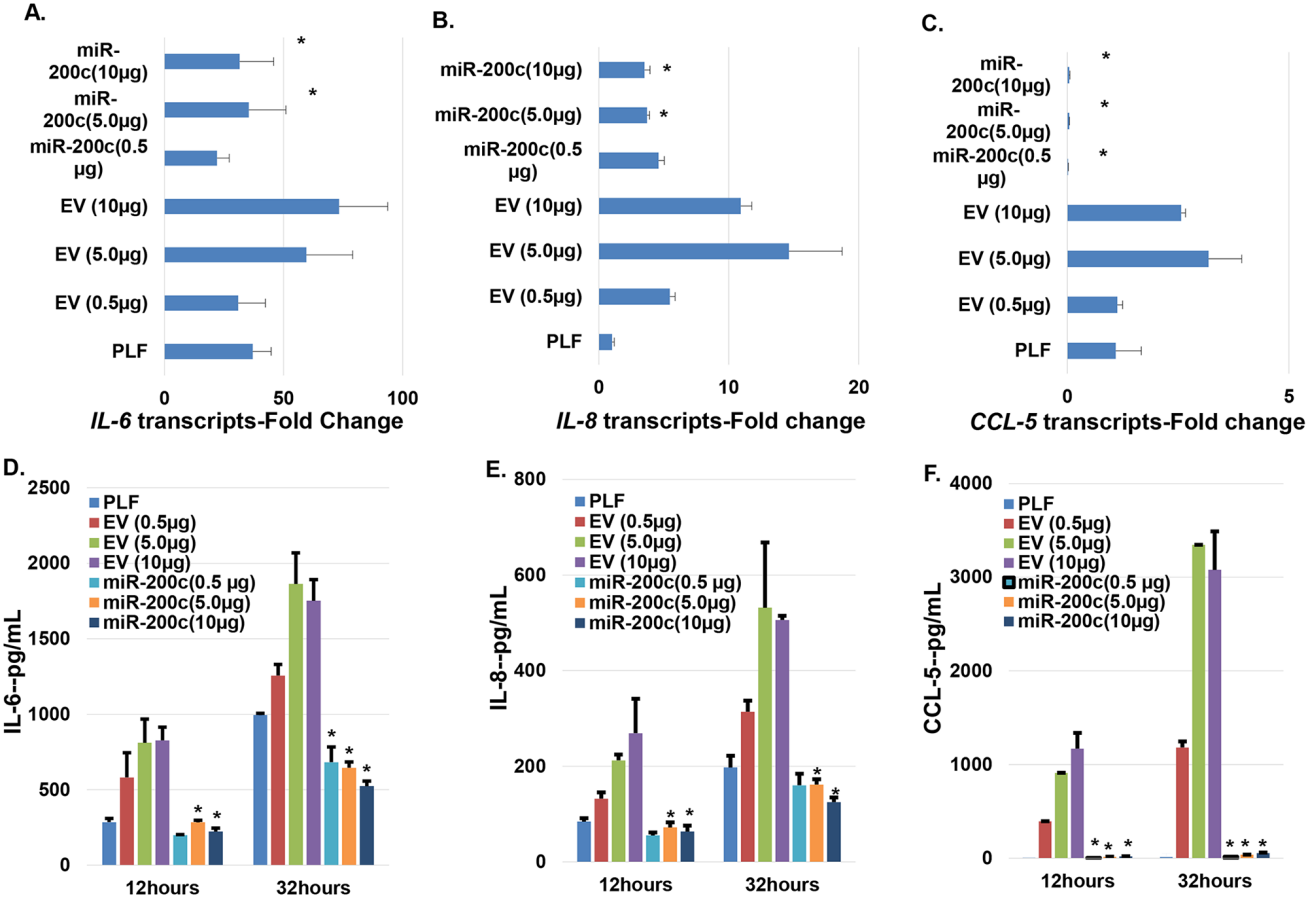
*miR-200c* delivered using PEI nanoparticles inhibits IL-6, IL-8, and CCL-5 in primary human periodontal ligament fibroblasts. **A-C**: The transcripts of IL-6 (**A**), IL-8 (**B**), and CCL-5 (**C**) in the cells with *miR-200c* or empty vector cultured in DMEM supplemented with LPS after 24 hours; **D** and **E**: the amounts of IL-6 (**D**), IL-8 (**E**), and CCL-5 (**F**) secreted by the cells with miR-200c or empty vector cultured in DMEM supplemented with LPS after 12 and 32 hrs, respectively. *: p<0.05 vs empty vector with the same amount.

After primary human periodontal ligament fibroblasts were treated with PEI-*miR-200c* at different concentrations, the cells were cultured using DMEM with LPS supplement (1μg/ml) for up to 32 hours. The transcripts levels of IL-8, IL-6 with PEI-*miR-200c* nanoplex treatment at 5 and 10μg/per well, and CCL-5 at all concentrations in human periodontal ligament cells were lower than that of controls with treatment using the empty vectors at the same concentrations after 24 hours (Fig 5A, 5B and 5C). Furthermore, the concentrations of IL-6, IL-8, and CCL-5 in the supernatant of cells with different treatments after 12 and 32 hours were also decreased after m*iR-200c* nanoplex treatments (Fig 5D, 5E and 5F). Similar to the transcript quantitation, the cell receiving PEI-empty vector at 5 and 10 μg produced higher concentrations of IL-6, IL-8, and CCL-5 compared to control cells without transfection and the increase was statistically significant after 32 hours. However, the protein levels of these mediators in cells treated with PEI-*miR-200c* were significantly lower than that of cells treated with same concentration of empty vectors. Also, the *miR-200c* cells had lower levels of IL-6 and IL-8 than control cells without transfection after 32 hours.

### *miR-200c* directly targets the *3’UTR* of IL-6, IL-8, and CCL-5

**Fig 6:**
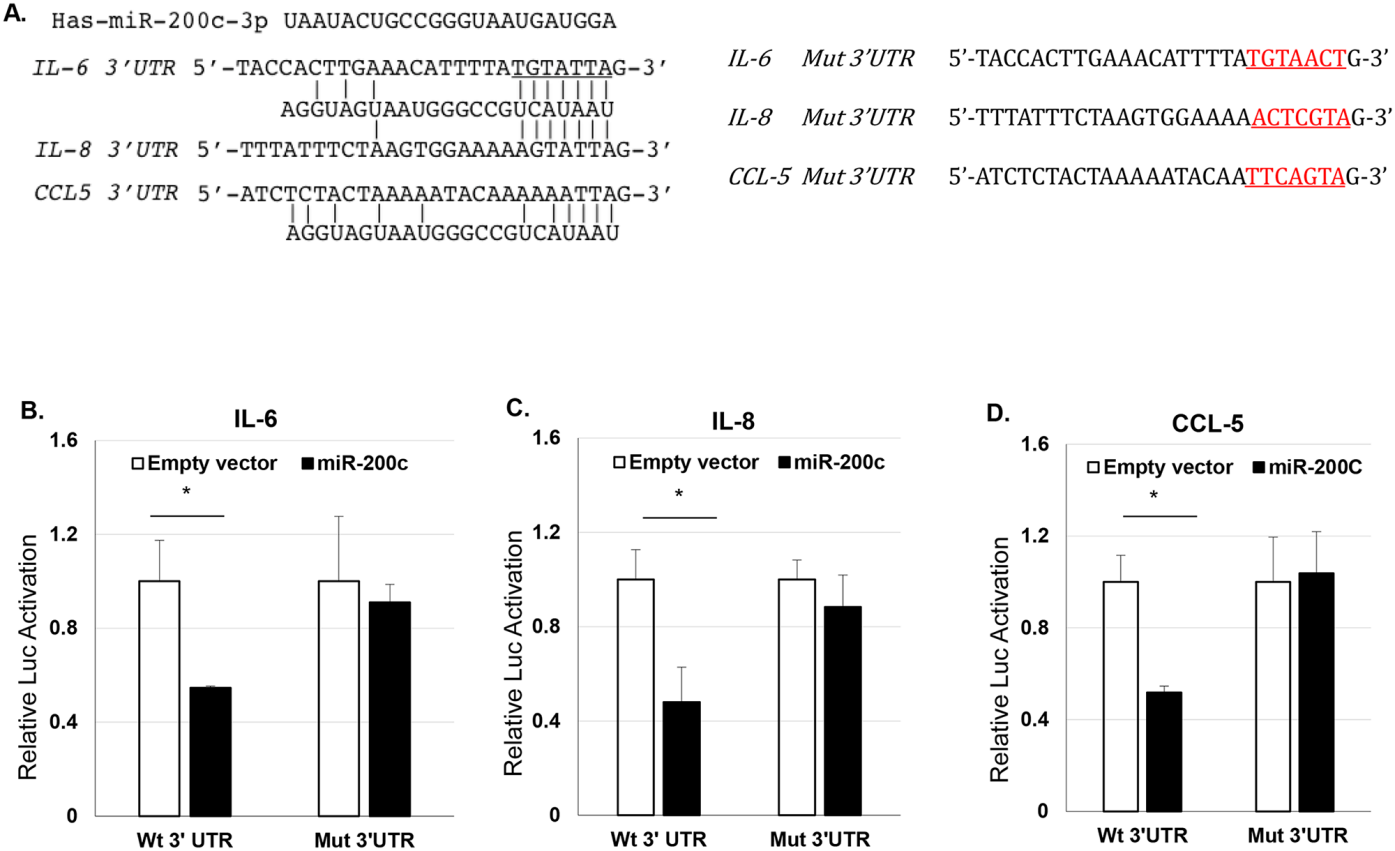
*miR-200c* directly targets the *3’UTR* of IL-6, IL-8, and CCL-5. **A**: The sequence and *miR-200c* binding region located in the *3’UTR* and mutated *3’UTR* of IL-6, IL-8, and CCL-5. **B-D**: Normalized luciferase activities of the *3’ UTR* IL-6, IL-8, and CCL-5-luciferase reporters and their *3’UTR*-mutated-luciferase reporters treated with empty vector or *miR-200c*. *: p<0.05.

To test if *miR-200c* directly targets these mediators the *3’UTR* sequence was cloned after the luciferase gene and luciferase activity was determined with and without *miR-200c* present. The sequence and *miR-200c* binding region located in the *3’UTR* of each mediator is shown in Fig 6A. *miR-200c* repressed luciferase activity from the IL-6, IL-8 and CCL-5 reporter constructs co-transfected in cells (Fig 6B, 6C and 6D). Normalized luciferase activity of the luciferase reporter with *3’ UTR* of IL-6, IL-8, and CCL-5 showed significantly lower with expression of *miR-200c* compared with the empty plasmid vector. However, there was no loss of luciferase activity when the *miR-200c* binding sequence was mutated in the *3’UTR* of IL-6, IL-8 and CCL-5 (Fig 6).

### The *miR-200c* inhibitor restore the activity of *miR-200c* to IL-6, IL-8, and CCL-5

**Fig 7:**
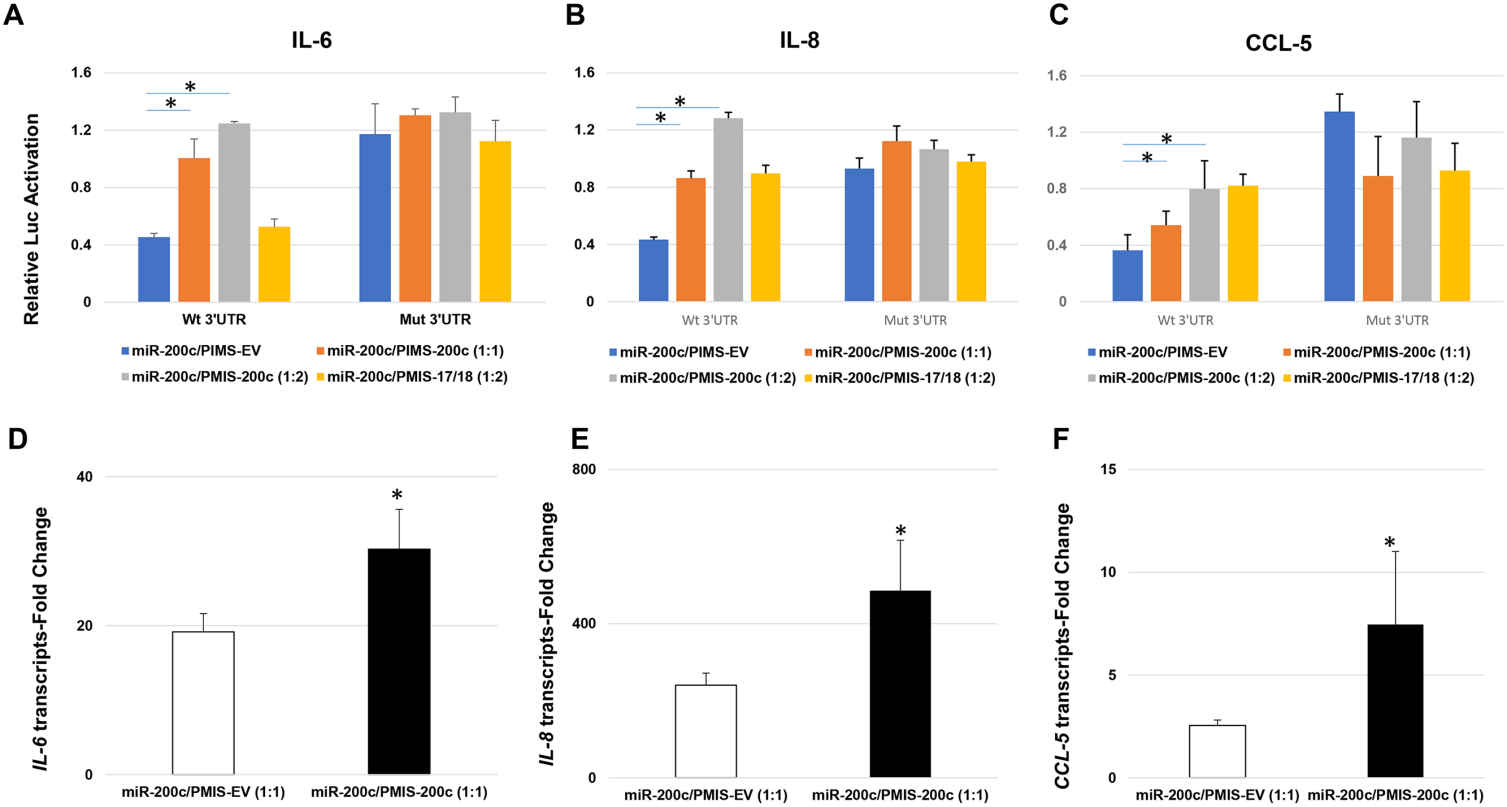
*PMIS-200c* reduces binding activity of *miR-200c* to the *3’UTR* of IL-6, IL-8, and CCL-5 and the function of *miR-200c*. **A-C**: Normalized luciferase activities of the *3’ UTR* IL-6, IL-8, and CCL-5-luciferase reporters and their *3’UTR*-mutated-luciferase reporters co-treated with *miR-200c* and *PMIS-EV* or PMIS-200c at different ratios of concentration. **D-F**: the transcripts of IL-6 (**D**), IL-8 (**E**), and CCL-5 (**F**) in the cells co-treated with *miR-200c* and *PMIS-EV* or *PMIS-200c* cultured in DMEM supplemented with LPS after 24 hours; *: p<0.05.

To further confirm if *miR-200c* directly targets *3’UTR* of these mediators, the luciferase activity inhibited by *miR-200c* was determined after treatment with a plasmid-base *miR* inhibitor system (*PMIS*) designed to bind *miR-200c* (*PMIS-200c*). *PMIS-200c* significantly increased luciferase activity from the luciferase reporter containing the *3’ UTR* of IL-6, IL-8, and CCL-5 treated with *miR-200c*, compared to *PMIS* designed to bind empty vector *(PMIS-EV)* or *miR-17/18 (PMIS-17/18)*. However, there was less change of luciferase activity when the mutated sequence in the *3’UTR* of IL-6, IL-8 and CCL-6 was used (Fig 7A, 7B and 7C). In addition, *PMIS-200c* also significantly increased the transcripts of IL-6, IL-8 and CCL-5 *in human periodontal ligament fibroblasts* with overexpression of *miR-200c* after *LPS* stimulation compared to *PMIS-EV*. These results further indicated the inhibitory effects of IL-6, IL-8 and CCL-5 mediated by *miR-200c* by targeting their *3’ UTRs*.

### *miR-200c* delivered using PEI improves osteogenic differentiation of human MSCs

**Fig 8:**
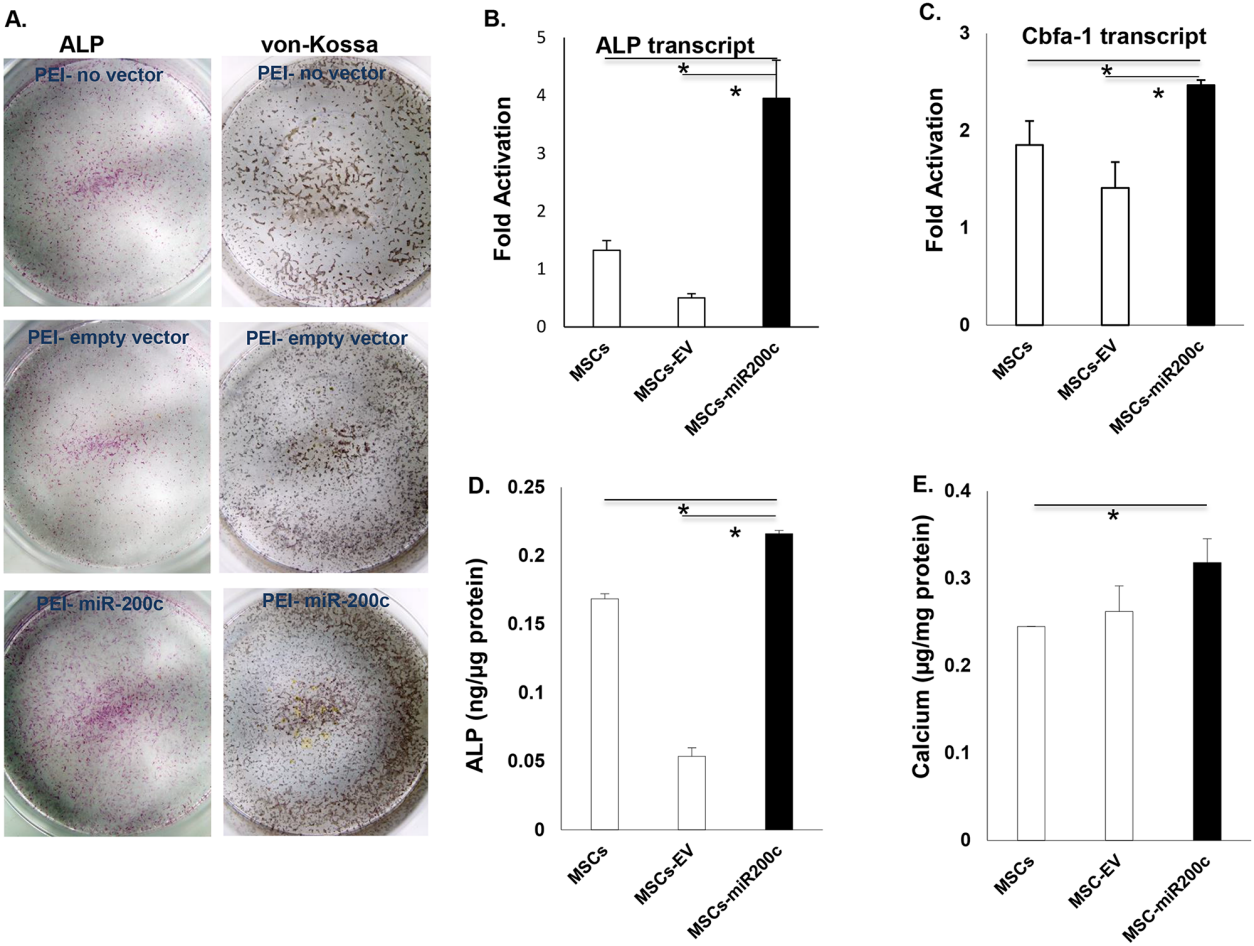
Enhancement of osteogenic differentiation of human bone marrow MSCs with overexpression of *miR-200c* using PEI nanoparticles. **A**: Images of ALP and von-Kossa staining in MSCs overexpressing *miR-200c*, one and two weeks after treatment with osteogenic medium. **B** and **C**: the transcripts of ALP (**B**) and Runx2 (**C**) in MSCs overexpressing *miR-200c*, one week after treatment with osteogenic medium. **D** and **E**: Quantitative measurement of ALP levels (**D**) and calcium content (**E**) in MSCs overexpressing *miR-200c*, one and two week after treatment with osteogenic medium. Each measurement was made in triplicate. *: p<0.05.

Human bone marrow MSCs were transfected using PEI-*miR-200c* or PEI-empty vector at 1.0 μg/per well, then the cells were subsequently cultured using DMEM supplemented with ascorbic acid and β-glycerophosphate for up to 2 weeks. ALP staining of MSCs showed mineralization effects with different treatment in culture dishes, 1 week after culture in osteogenic medium (Fig 8A). Staining was observed in both controls, including MSCs with and without treatment with the PEI-empty vector. PEI-*miR-200c* transfected cells showed stronger ALP staining than that in controls. von-Kossa staining was observed in MSCs with and without treatment with the PEI-empty vector. The von-Kossa staining was darker in the MSCs transfected with *miR-200c*. Quantitatively, the transcripts of ALP (Fig 8B) and *Runx2* (Fig 8C) were significantly increased in the cells treated with PEI-*miR-200c*, compared to controls including MSCs treated with PEI, with or without empty vector. In addition, the ALP concentration (Fig 8D) and calcium content (Fig 8E) in the MSCs transfected with *miR-200c* were increased after 2 weeks in culture.

## Discussion

In this study we observed that, for the first time, overexpression of *miR-200c* effectively represses multiple proinflammatory mediators, including IL-6, IL-8 and CCL-5, in a human preosteoblast cell line. Our Gene analysis indicated that *miR-200c* may directly target the *3’UTR* of these mediators. Overexpression of *miR-200c* may also promote *OPG* and improve osteogenic differentiation in this cell line. Furthermore, our studies demonstrated that plasmid DNA containing *miR-200c* can be effectively delivered using a non-viral delivery system. *miR-200c* delivered using PEI nanoparticles inhibited IL-6, IL-8, and CCL-5 in primary human periodontal ligament fibroblasts and improved osteogenic differentiation of primary human bone marrow MSCs. These data strongly suggest that *miR-200c* may potentially be used to repress periodontitis-associated bone resorption and restore the periodontal bone defects by improving bone formation and modulating imbalance and dysregulation of proinflammatory mediators.

While *miR-200c* is downregulated in gingival tissues of periodontitis patient, its function and underlying mechanism(s) in this chronic inflammatory disease are less understood. Several publications have suggested that *miR-200c* may participate in the regulation of inflammation. Rokavec et al. reported that *miR-200c* suppression may direct the constitutive activation of inflammatory signaling circuit in transformation and tumorigenesis [25]. Wendlandt et al. demonstrated that *miR-200c* may reduce NF-kB activation by modifying TLR-4 signaling through the MyD88-dependent pathway [26]. miR-200c may also reduce IL-8 expression by targeting IKBKB in NF-kB signal pathway [32]. Another report indicated that *miR-200c* may target an NF-κB up-regulated TrkB/NTF3 autocrine signaling loop in breast tumors [27]. However, this is the first study to investigate the anti-inflammatory activity of *miR-200c* in periodontitis and related bone metabolism. In this study, although the effect mediated by *miR-200c* on the regulation of the p65/p50 subunits of NF-kB was not observed *(data not shown)*, we have shown that, *miR-200c* effectively inhibits IL-8 expression in human preosteoblasts and periodontal ligament fibroblast under the stimulation of a bacterial endotoxin by binding to the IL-8 *3’UTR*. Moreover, we demonstrated that *miR-200c* effectively reduces IL-6 and CCL-5 expression in human preosteoblasts and periodontal ligament fibroblasts. The reporter gene analysis also demonstrated that *miR-200c* effectively targets the *3’UTR* of IL-6 and CCL-5. As IL-6, IL-8 and CCL-5 major proinflammatory mediators having critical roles in inflammation, these results strongly suggest that *miR-200c* possesses a powerful capacity to reduce inflammation via post-transcriptional regulation of these proinflammatory mediators.

We observed that overexpression of *miR-200c* upregulated OCN and calcium content in human preosteoblast cells. Furthermore, we demonstrated that *miR-200c* delivered using PEI nanoparticles promotes increased ALP, Runx2, and calcium content in primary human MSCs. While *miR-200c* can participate in stem cell proliferation and differentiation [24], this is the first study that demonstrates the potential function of *miR-200c* to improve osteogenic differentiation and bone regeneration. This function may be accomplished by inhibiting Noggin, an antagonist of BMP signals. We have previously reported that *miR-200c* targets Noggin *3’UTR* and down-regulates Noggin expression in dental epithelial cells [28]. Noggin is a secreted protein that binds and inactivates a number of BMPs, including BMP-2, 7. Noggin suppression has been demonstrated to promote *BMP*-induced bone regeneration *in vitro* and *in vivo* [33, 34]. In this study, we also observed *miR-200c* inhibition of Noggin expression in human bone marrow MSCs (data not shown). Additionally, proinflammatory mediators have been demonstrated to impair bone formation by reducing differentiation of osteoblasts and their progenitor cells [17, 18]. Inhibition of proinflammatory mediators also partially explains the function of *miR-200c* on enhancing bone formation.

Although non-viral gene delivery systems have shown promise as alternative approaches of recombinant viral vectors, nanoparticles are the only non-viral vectors that can provide a targeted intracellular delivery with controlled release properties. Nanoparticles can serve as a local drug delivery system to oral mucosa [35]. PEI nanoparticles have been used as a non-viral vector for gene delivery due to their “proton-sponge” effect and high transfection efficiency. Our previous studies have successfully delivered plasmid DNA using PEI nanoparticles. We demonstrated that N/P ratios significantly influence the size, surface charge, transfection efficiency, and cytotoxicity of PEI nanoplexes. Our previous studies have also shown that PEI-pDNA {encoding for platelet derived growth factor-B (PDGF-B)} nanoplexes can induce significantly higher bone regeneration in calvarial rat defects [30]. In this study we showed that PEI can effectively deliver *miR-200c* into primary human periodontal ligament fibroblasts and bone marrow MSCs, demonstrating the feasibility of transfecting *miR-200c* using PEI nanoparticles. These results, along with our previous *in vivo* studies, strongly suggest that PEI nanoparticles may potentially be used as a delivery system to transfect *miR-200c* for clinical application purposes. In addition, we observed that the up-regulated content of *miR-200c* expression is dose-dependent according to PEI-*miR-200c* nanoplex treatment. Thus, we can maximize the effects of *miR-200c* by optimizing the PEI-*miR-200c* nanoplex concentration and its expression level.

In this study, we observed that the secreted amounts of IL-6, IL-8, and CCL-5 increased with the dose of transfection of plasmid DNA, which indicates that the cellular inflammation response and inhibitory effects can be mediated by *miR-200c* in response to the stimulation by bacterial endotoxin. This is probably caused by the innate immune system of the cells that can recognize nucleic acids after transfection. It has been demonstrated that after plasmid DNA is detected by endosomal toll-like receptors, including TLR3, TLR7, and TLR8, and cytoplasmic RIG-I and MDA5, endosomal TLR9 and cytoplasmic DAI may bind the DNA, resulting in the activation of NF-kB and interferon regulatory factor transcription factors [36]. Therefore, in order to develop a plasmid *miR-200c* based approach for anti-inflammation, an optimal transfection of *miR-200c* to limit plasmid DNA-induced innate immune response is necessary. Besides periodontitis, IL-6, IL-8, and CCL-5 as major proinflammatory mediators they also play critical roles in many inflammation-related diseases, including osteoarthritis and Parkinson’s disease. Thus, the inhibitory effects mediated by *miR-200c* indicate that this *miR* may be potentially developed into a therapeutic tool for these diseases.

## References

[1] Eke PI, Dye BA, Wei L, Thornton-Evans GO, Genco RJ. CDC Periodontal Disease Surveillance workgroup: James Beck (University of North Carolina, Chapel Hill, USA), Gordon Douglass (Past President, American Academy of Periodontology), Roy Page (University of Washin. Prevalence of periodontitis in adults in the United States: 2009 and 2010. J Dent Res. 2012; 91: 914–1920. 22935673 10.1177/0022034512457373

[2] Papapanou PN, Tonetti MS. Diagnosis and epidemiology of periodontal osseous lesions. Periodontol. 2000; 22: 8–21. 10.1034/j.1600-0757.2000.2220102.x 11276518

[3] Müller HP, Ulbrich M. Alveolar bone levels in adults as assessed on panoramic radiographs. (I) Prevalence, extent, and severity of even and angular bone loss. Clin Oral Investig. 2005; 9: 98–104 15834742 10.1007/s00784-005-0303-x

[4] Armas J, Culshaw S, Savarrio L. Treatment of peri-implant diseases: a review of the literature and protocol proposal. Dent Update. 2013; 40:472–480. 23971346 10.12968/denu.2013.40.6.472

[5] Jeffcoat MK. Osteoporosis: a possible modifying factor in oral bone loss. Ann Periodontol. 1998; 3: 312–321 9722715 10.1902/annals.1998.3.1.312

[6] Sidiropoulou-Chatzigiannis S, Kourtidou M, Tsalikis L. The effect of osteoporosis on periodontal status, alveolar bone and orthodontic tooth movement. A literature review. J Int Acad Periodontol. 2007; 9: 77–84 17715839

[7] Khosla S, Bilezikian JP, Dempster DW, Lewiecki EM, Miller PD, Neer RM, et al Benefits and risks of bisphosphonate therapy for osteoporosis. J Clin Endocrinol Metab. 2012; 97: 2272–82. 10.1210/jc.2012-1027 22523337

[8] Darveau RP. (2010) Periodontitis: a polymicrobial disruption of host homeostasis. Nat Rev Microbiol. 2010; 8: 481–490. 10.1038/nrmicro2337 20514045

[9] Di Benedetto A, Gigante I, Colucci S, Grano M. Periodontal disease: linking the primary inflammation to bone loss. Clin Dev Immunol. 2013; 2013:503754 10.1155/2013/503754 23762091 PMC3676984

[10] Hajishengallis G, Krauss JL, Liang S, McIntosh ML, Lambris JD. Pathogenic microbes and community service through manipulation of innate immunity. Adv Exp Med Biol. 2012: 946: 69–85. 10.1007/978-1-4614-0106-3_5 21948363 PMC3214273

[11] Herath TD, Darveau RP, Seneviratne CJ, Wang CY, Wang Y, Jin L. Tetra- and penta-acylated lipid A structures of Porphyromonas gingivalis LPS differentially activate TLR4-mediated NF-κB signal transduction cascade and immuno-inflammatory response in human gingival fibroblasts. PLoS One. 2013; 8:e58496 10.1371/journal.pone.0058496 23554896 PMC3595299

[12] Graves DT, Oates T, Garlet GP. Review of osteoimmunology and the host response in endodontic and periodontal lesions. J Oral Microbiol. 2011; 17:3 10.3402/jom.v3i0.5304 PMC3087239 21547019

[13] Yang N, Wang G, Hu C, Shi Y, Liao L, Shi S, et al Tumor necrosis factor α suppresses the mesenchymal stem cell osteogenesis promoter miR-21 in estrogen deficiency-induced osteoporosis. J Bone Miner Res. 2013; 28: 559–573. 10.1002/jbmr.1798 23074166

[14] Lacey DC, Simmons PJ, Graves SE, Hamilton JA. Proinflammatory cytokines inhibit osteogenic differentiation from stem cells: implications for bone repair during inflammation. Osteoarthritis Cartilage. 2009; 17: 735–742. 10.1016/j.joca.2008.11.011 19136283

[15] Hikiji H, Shin WS, Koizumi T, Takato T, Susami T, Koizumi Y, et al Peroxynitrite production by TNF-alpha and IL-1beta: implication for suppression of osteoblastic differentiation. Am J Physiol Endocrinol Metab. 2000; 278: E1031–1037. 10827005 10.1152/ajpendo.2000.278.6.E1031

[16] Wang L, Zhao Y, Shi S. Interplay between mesenchymal stem cells and lymphocytes: implications for immunotherapy and tissue regeneration. J Dent Res. 2012; 91: 1003–1010 10.1177/0022034512460404 22988011 PMC3490280

[17] Chang J, Wang Z, Tang E, Fan Z, McCauley L, Franceschi R, et al Inhibition of osteoblastic bone formation by nuclear factor-kappaB. Nat Med. 2009; 15: 682–689 10.1038/nm.1954 19448637 PMC2768554

[18] Chang J, Liu F, Lee M, Wu B, Ting K, Zara JN, et al NF-κB inhibits osteogenic differentiation of mesenchymal stem cells by promoting β-catenin degradation. Proc Natl Acad Sci U S A. 2013; 110: 9469–9474. 10.1073/pnas.1300532110 23690607 PMC3677422

[19] Singh RP, Massachi I, Manickavel S, Singh S, Rao NP, Hasan S, et al The role of miRNA in inflammation and autoimmunity. Autoimmun Rev. 2013; 12: 1160–1165. 10.1016/j.autrev.2013.07.003 23860189

[20] Plank M, Maltby S, Mattes J, Foster PS. Targeting translational control as a novel way to treat inflammatory disease: the emerging role of microRNAs. Clin Exp Allergy. 2013; 43: 981–999. 10.1111/cea.12170 23957346

[21] Lian JB, Stein GS, van Wijnen AJ, Stein JL, Hassan MQ, Gaur T, et al MicroRNA control of bone formation and homeostasis. Nat Rev Endocrinol. 2012; 8: 212–227. 10.1038/nrendo.2011.234 22290358 PMC3589914

[22] Katoh Y, Katoh M. Hedgehog signaling, epithelial-to-mesenchymal transition and miRNA (review). Int J Mol Med. 2008; 22: 271–275. 18698484

[23] Huang HN, Chen SY, Hwang SM, Yu CC, Su MW, Mai W, et al miR-200c and GATA binding protein 4 regulate human embryonic stem cell renewal and differentiation. Stem Cell Res. 2014; 12: 338–353. 10.1016/j.scr.2013.11.009 24365599

[24] Stoecklin-Wasmer C, Guarnieri P, Celenti R, Demmer RT, Kebschull M, Papapanou PN. MicroRNAs and their target genes in gingival tissues. J Dent Res. 2012; 91: 934–40 22879578 10.1177/0022034512456551 PMC3446831

[25] Rokavec M, Wu W, Luo JL. IL6-mediated suppression of miR-200c directs constitutive activation of inflammatory signaling circuit driving transformation and tumorigenesis. Mol Cell. 2012; 45: 777–789. 10.1016/j.molcel.2012.01.015 22364742 PMC3319241

[26] Wendlandt EB, Graff JW, Gioannini TL, McCaffrey AP, Wilson ME. The role of microRNAs miR-200b and miR-200c in TLR4 signaling and NF-κB activation. Innate Immun. 2012; 18: 846–855. 10.1177/1753425912443903 22522429 PMC3733339

[27] Howe EN, Cochrane DR, Cittelly DM, Richer JK. miR-200c targets a NF-κB up-regulated TrkB/NTF3 autocrine signaling loop to enhance anoikis sensitivity in triple negative breast cancer. PLoS One. 2012; 7:e49987 10.1371/journal.pone.0049987 23185507 PMC3503774

[28] Cao H, Jheon A, Li X, Sun Z, Wang J, Florez S, et al The Pitx2:miR-200c/141:noggin pathway regulates Bmp signaling and ameloblast differentiation. Development. 2013; 140: 3348–3359. 10.1242/dev.089193 23863486 PMC3737717

[29] Cao H, Yu W, Li X, Wang J, Gao S, Holton NE, et al A new plasmid-based microRNA inhibitor system that inhibits microRNA families in transgenic mice and cells: A potential new therapeutic reagent. Gene Ther. 2016; 3 2 10.1038/gt.2016.22 PMC6946376 27383391

[30] Elangovan S., D'Mello S.R., Hong L., Ross R.D., Allamargot C., Dawson D.V., et al The enhancement of bone regeneration by gene activated matrix encoding for platelet derived growth factor. Biomaterials 2014; 35: 737–747 10.1016/j.biomaterials.2013.10.021 24161167 PMC3855224

[31] Borgwardt DS, Martin AD, Van Hemert JR, Yang J, Fischer CL, Recker EN, et al Histatin 5 binds to Porphyromonas gingivalis hemagglutinin B (HagB) and alters HagB-induced chemokine responses. Sci Rep. 2014; 4: 3904 10.1038/srep03904 24473528 PMC3912440

[32] Chuang TD, Khorram O. miR-200c regulates IL8 expression by targeting IKBKB: a potential mediator of inflammation in leiomyoma pathogenesis. PLoS One. 2014; e95370 10.1371/journal.pone.0095370 24755559 PMC3995706

[33] Stottmann RW, Anderson RM, Klingensmith J. The BMP antagonists Chordin and Noggin have essential but redundant roles in mouse mandibular outgrowth. Dev Biol. 2001; 240: 457–273. 11784076 10.1006/dbio.2001.0479

[34] Heliotis M, Tsiridis E. Suppression of bone morphogenetic protein inhibitors promotes osteogenic differentiation: therapeutic implications. Arthritis Res Ther. 2008; 10: 115 10.1186/ar2467 18710600 PMC2575635

[35] Holpuch AS, Hummel GJ, Tong M, Seghi GA, Pei P, Ma P, et al Nanoparticles for local drug delivery to the oral mucosa: proof of principle studies. Pharm Res. 2010: 27: 1224–1236. 10.1007/s11095-010-0121-y 20354767 PMC2883929

[36] Mogensen TH. Pathogen recognition and inflammatory signaling in innate immune defenses. Clin Microbiol Rev. 2009; 22: 240–273 10.1128/CMR.00046-08 19366914 PMC2668232

