# Correction: *MicroRNA-200c* Represses IL-6, IL-8, and CCL-5 Expression and Enhances Osteogenic Differentiation

**DOI:** 10.1371/journal.pone.0169381

**Published:** 2016-12-29

**Authors:** Liu Hong, Thad Sharp, Behnoush Khorsand, Carol Fischer, Steven Eliason, Aliasger K. Salem, Adil Akkouch, Kim Brogden, Brad A. Amendt

The sixth author’s name is spelled incorrectly. The correct name is: Aliasger K Salem. The correct citation is: Hong L, Sharp T, Khorsand B, Fischer C, Eliason S, Salem AK, et al. (2016) *MicroRNA-200c* Represses IL-6, IL-8, and CCL-5 Expression and Enhances Osteogenic Differentiation. PLoS ONE 11(8): e0160915. doi:10.1371/journal.pone.0160915
